# Thermal Stability and Barrier Properties of Polyamide 6 Reinforced by Carbazole Based Copolymerization

**DOI:** 10.3390/polym18050559

**Published:** 2026-02-25

**Authors:** Yong Yi, Jianlin Li, Wenzhi Wang, Chunhua Wang, Yuejun Liu

**Affiliations:** 1 National & Local Joint Engineering Research Center for Advanced Packaging Material and Technology, Hunan University of Technology, Zhuzhou 412007, China; yiyong@stu.hut.edu.cn (Y.Y.); lijianlin@stu.hut.edu.cn (J.L.); wangwenzhi@hut.edu.cn (W.W.); 2 Key Laboratory of Advanced Packaging Materials and Technology of Hunan Province, Hunan University of Technology, Zhuzhou 412007, China; 3 Zhuzhou Times New Material Technology Co., Ltd., Zhuzhou 412007, China

**Keywords:** carbazole group, thermal stability, dimensional stability, barrier properties

## Abstract

Polyamide 6 (PA6) is limited in its application in precision and high-temperature fields due to its high moisture absorption, low heat resistance, and poor barrier properties. To overcome these intrinsic deficiencies, a rigid 9-(carboxyphenyl)carbazole-based diacid monomer (CzIPA) was incorporated into the PA6 backbone via one-step melt polycondensation. Structural analyses confirmed successful copolymer formation and effective modulation of hydrogen-bonding interactions and chain rigidity. The introduction of the bulky carbazole units markedly enhanced the thermal and physical properties of PA6. The glass transition temperature increased by up to 35.5 °C, while the maximum decomposition temperature rose by 23.8 °C, reflecting the reduced chain mobility and strengthened thermal resistance. The decreased amide-group density led to a 15% reduction in water absorption, improving dimensional stability. The Young’s modulus, flexural strength, and flexural modulus of the prepared copolymers were significantly improved compared to PA6, while the toughness was slightly reduced. Furthermore, oxygen and water-vapor permeabilities were simultaneously reduced by 30–35%, attributed to restricted diffusion pathways in the modified microstructure. Despite the increased rigidity, the copolymers maintained good melt processability with clear shear-thinning behavior. This study demonstrates CzIPA copolymerization as an efficient structural design strategy for producing high-performance PA6 materials with enhanced thermal stability, lower hygroscopicity, and superior barrier properties.

## 1. Introduction

Polyamide 6 (PA6), is one of the most important engineering thermoplastics, widely used in automotive parts, electronic devices, packaging films, textiles [1], and optical materials [2]. Owing to its excellent mechanical strength, chemical resistance, and processability, PA6 has become a preferred material for high-performance applications [3]. However, despite these advantages, PA6 still faces critical limitations that hinder its broader application. The strong polarity of the amide groups leads to high moisture absorption, which causes dimensional instability, degradation of mechanical properties. In addition, the relatively low glass transition temperature (*Tg*) [4] and poor heat distortion resistance limit its use under high-temperature or humid environments [5,6]. Therefore, improving the dimensional stability and thermal resistance of PA6 has become an essential direction for further material optimization and industrial application.

To overcome these shortcomings, various modification strategies have been developed to enhance the performance of PA6 [7,8]. By blending PA6 with polymers or inorganic fillers possessing low moisture uptake and high thermal resistance, its dimensional stability [9], thermal properties, and barrier performance can be effectively improved [10]. Common blending components include polyethylene [11], polyesters [12], and inorganic nanofillers [13] such as silica [14], montmorillonite [15], carbon nanotubes [16], and graphene [17]. These materials effectively reduce moisture uptake, enhance thermal stability, and improve mechanical strength through physical entanglement and interfacial reinforcement, thereby enhancing the overall dimensional and structural stability of PA6-based materials. However, PA6 is a polar polyamide, its compatibility with non-polar or weakly polar polymers is often poor, resulting in phase separation and heterogeneous morphologies. This lack of strong interfacial adhesion weakens the mechanical integrity and long-term stability of the blends.

In contrast to blending, copolymerization provides a more effective and molecular designing strategy to modify PA6 [18,19]. Through the incorporation of comonomer units into the PA6 backbone, copolymerization enables precise control over the polymer chain structure, segmental mobility, and intermolecular interactions [20,21]. This approach not only improves compatibility between different segments but also allows the introduction of functional groups that can tailor the thermal, mechanical, and barrier properties of PA6. Wang et al. [22]. demonstrated that incorporating comonomer units into PA6 disrupts chain regularity in a controlled manner, facilitating the reorganization of hydrogen-bond networks and thereby enhancing stiffness, strength, and thermal stability. Moreover, introducing rigid aromatic structures or bulky cyclic groups into the PA6 main chain can effectively restrict chain motion, leading to improved heat resistance and dimensional stability [23,24,25]. Zhang et al. [26]. showed that introducing rigid aromatic imide units into the PA6 backbone effectively alters chain packing and reduces crystallinity, thereby restricting segmental mobility and increasing *Tg*, which collectively enhance the thermal stability and overall performance of the copolymer.

Carbazole-based monomers have recently attracted considerable attention due to their rigid conjugated aromatic structures [27], high thermal stability [28], and strong intermolecular interactions [29,30]. The incorporation of carbazole moieties into polymer backbones can significantly enhance chain rigidity, reduce segmental mobility, and improve thermal and dimensional stability [31]. Tang et al. [32]. showed that incorporating carbazolyl units into organic radicals enhances structural rigidity, improves stability, and tunes photophysical properties, thereby demonstrating their effectiveness as versatile building blocks for advanced polymer systems. Furthermore, the nitrogen atom within the carbazole ring can form hydrogen bonds or polar interactions with adjacent amide groups, potentially improving cohesive energy and gas barrier performance [33,34]. Yang et al. [35]. demonstrated that introducing 4,4′-bis(N-carbazolyl)-1,1′-biphenyl as a branched linker into the poly(p-terphenyl piperidinium) backbone effectively enhances the conductivity, dimensional stability, and chemical robustness of anion exchange membranes, thereby improving their performance.

In this study, a novel PA6-based copolymer was designed and synthesized using 9-(carboxyphenyl)carbazole-based diacid monomer (CzIPA) as a monomer. The introduction of this rigid, planar π-conjugated unit into PA6 chains significantly enhanced the thermal stability of the copolymers and reduced hydrogen bond density, while largely retaining the intrinsic mechanical properties of PA6. Improved the gas barrier performance of nylon 6 against oxygen and water vapor. The structure of the copolymers was confirmed by NMR and FTIR spectroscopy, and their processing performance was systematically investigated using capillary and rotational rheometry. This work not only broadens the application scope of carbazole-based derivatives in PA6 systems but also provides new insights into the molecular design of high-performance engineering plastics.

## 2. Experimental

### 2.1. Materials

3,6-Dibromocarbazole, 4-methylphenylboronic acid, tetrakis (triphenylphosphine) palladium [Pd(PPh_3_)_4_], 5-aminoisophthalic acid dimethyl ester, and 2,2′-dibromobiphenyl were purchased from Shanghai Macklin Biochemical Co., Ltd. (purity ≥ 98.5%). Tetrahydrofuran (THF) and sodium bicarbonate (NaHCO_3_) were supplied by Tianjin Kemiou Chemical Reagent Co., Ltd. (analytical grade). Decamethylenediamine was obtained from Hebei Jinghua Chemical Co., Ltd. (purity ≥ 99%), and ε-caprolactam was purchased from DSM Nanjing Oriental Chemical Co., Ltd. (purity ≥ 99%). The carbazole-based dicarboxylic acid monomer, deionized water, and high-purity nitrogen (≥99.9%) were prepared in-house. All reagents were used as received unless otherwise noted. Caprolactam, purity 99.5, Nanjing DSM Chemicals Co., Ltd. Decanediamine, purity 99.5, Shanghai Macklin Biochemical Co., Ltd.

### 2.2. Preparation of Carbazolylic Acid

A novel side-chain carbazole-based dicarboxylic acid was synthesized via a Suzuki–Miyaura coupling reaction [36]. Specifically, 209.2 g (1.0 mol) of 5-aminoisophthalic acid dimethyl ester, 312.0 g (1.0 mol) of 2,2′-dibromobiphenyl, 57.8 g (0.05 mol) of Pd(PPh_3_)_4_, and 106.0 g (1.0 mol) of Na_2_CO_3_ were sequentially added into a 5000 mL three-neck round-bottom flask. Subsequently, 1707.8 g of tetrahydrofuran (THF) was introduced as the solvent. The mixture was stirred under a nitrogen atmosphere and refluxed at 70 °C for 24 h. After completion, the reaction mixture was filtered and dried to obtain the product 5-(9H-Carbazol-9-yl)isophthalic acid (CzIPA).

### 2.3. Preparation of Nylon Salts

The CzIPA was placed in a glass reactor, followed by the addition of an appropriate amount of deionized water. The mixture was mechanically stirred until a homogeneous solution was obtained. The temperature was raised to approximately 80 °C, after which an aqueous solution of decamethylenediamine was slowly added dropwise. The molar ratio of dicarboxylic acid to diamine was maintained at approximately 1: (1.01–1.02). The reaction was conducted under a nitrogen atmosphere with continuous stirring, and the temperature was gradually increased until the system became clear. Subsequently, the pH of the nylon salt solution was adjusted, and when the pH reached 7.2–7.8, the reaction was considered complete. The mixture was then kept at constant temperature for 0.5 h and cooled to obtain the nylon salt solution.

### 2.4. Synthesis of PA6/CzIPA Copolymer

**Figure 1:**
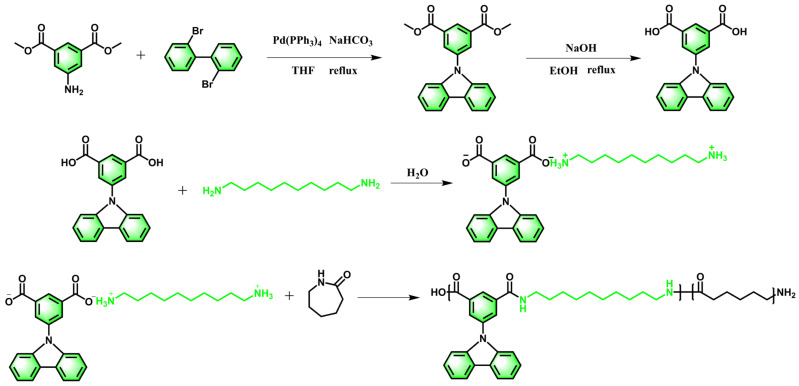
Synthetic routes of CzIPA and PA6 copolymers.

A predetermined amount of CzIPA, decamethylenediamine, ε-caprolactam, and deionized water was added to a high-temperature, high-pressure autoclave. Nitrogen was introduced to 0.3 MPa at room temperature and then released to atmospheric pressure. This purging process was repeated 3–5 times, followed by sealing the reactor under 0.2 MPa nitrogen. The reactor was then heated, and stirring was initiated when the temperature reached 80 °C. The temperature was further increased to 220 °C, at which point the system pressure reached approximately 1.8 MPa. The temperature and pressure were maintained for about 2 h to promote the hydrolytic ring-opening of ε-caprolactam and partial condensation between the dicarboxylic acid and diamine. Subsequently, the temperature was increased to 250 °C while the steam was gradually released, reducing the internal pressure to atmospheric within 2 h. A vacuum system was then connected, and the pressure was gradually decreased from atmospheric to −0.07 MPa over 1.5 h. The system was maintained at 250 °C and −0.07 MPa for an additional 0.5 h to facilitate further polycondensation and achieve higher molecular weight. After the vacuum was stopped, nitrogen was introduced to restore atmospheric pressure, and stirring was terminated. The bottom valve of the reactor was opened, and a small amount of nitrogen was introduced to extrude the molten polymer from the reactor. The extrudate was cooled in a water bath, pelletized, and then extracted in water at 100 °C for 72 h. Finally, the copolymer resin was vacuum-dried until the moisture content was below 0.1%. A schematic of the reaction process is shown in Figure 1. A series of copolymer resins containing different carbazole group contents were prepared by varying the proportion of nylon salt monomers, as listed in Table S1. The weight fraction of carbazole-based nylon salt was adjusted to 5%, 10%, 15%, 20%, and 30%, and the corresponding copolymers were designated as C5, C10, C15, C20, and C30, respectively.

### 2.5. Measurements

Gel permeation chromatography (GPC) was employed to analyze the molecular weight distribution of the polymers. Five mg of polyamide was dissolved in 1,1,1,3,3,3-hexafluoroisopropanol (HFIP) and then filtered through a 0.45 μm PTFE filter. To mitigate the polyelectrolyte effect of the nylon material, a small amount of sodium trifluoroacetate was added to the solvent. A calibration curve was constructed using PMMA as the standard, with the column temperature maintained at 40 °C. HFIP was used as the mobile phase, and the flow rate was set to 1 mL·min^−1^.

Fourier-transform infrared (FTIR) spectra were recorded on a Nicolet IS 10 spectrometer equipped with an attenuated total reflection (ATR) accessory.

^1^H and ^13^C nuclear magnetic resonance (NMR) spectra were obtained on a Bruker ARV-400 spectrometer using deuterated dimethyl sulfoxide (DMSO-d6) as solvent and tetramethyl silane (TMS) as an internal standard.

Thermal transitions were analyzed using a Netzsch DSC 204 F1 instrument under nitrogen atmosphere. Heating and cooling scans were performed at a rate of 10 °C/min.

Dynamic mechanical properties were evaluated using a DMTA-IV dynamic mechanical thermal analyzer (Rheometric Scientific, USA). Measurements were carried out from −100 °C to 150 °C at a heating rate of 3 °C/min with a frequency of 1 Hz.

Thermal decomposition behavior was measured on a Mettler Toledo TGA/DSC1 1100 SF instrument under nitrogen atmosphere. Samples were heated from 25 °C to 850 °C at a rate of 10 °C/min with a nitrogen flow rate of 50 mL/min.

Circular discs (25 mm diameter, 1 mm thickness) were prepared by compression molding. Strain and frequency sweeps were performed on a DMA 303 Eplexor rotational rheometer. Strain sweeps were conducted at 230 °C with a frequency of 6.28 rad/s over a strain range of 0.05–100%. Frequency sweeps were performed at 230, 240, 250, and 260 °C with a fixed strain of 1%, covering the frequency range of 0.05–100 rad/s.

One-dimensional WAXD patterns were collected on a Bruker D8 Discover diffractometer with Cu Kα radiation (λ = 0.154 nm). The operating voltage and current were set to 40 kV and 40 mA, respectively. The scanning range was 2θ = 5~40°.

Two-dimensional small-angle X-ray scattering (SAXS) measurements were carried out on a Xeuss 3.0 platform (Xenocs, Sassenage, France). The incident beam was generated using a Cu Kα radiation source (Genix3D, λ = 0.15418 nm). The sample-to-detector distances for SAXS was 530 mm, respectively, with an acquisition time of 300 s.

Dumbbell-shaped specimens were prepared by injection molding and conditioned at 23 °C and 50% RH for 48 h prior to testing. Tensile properties were determined according to GB/T 1040-2008 using an ETM 502 B-EX universal testing machine at a crosshead speed of 50 mm/min (Type 1A, 150 × 10 × 4 mm). Impact strength was evaluated according to GB/T 1043-2008 using a 7.5 J pendulum hammer (Type 1A, 80 × 10 × 4 mm). Flexural properties were measured in accordance with GB/T 9341-2008 at a crosshead speed of 2 mm/min (Type 1A, 80 × 10 × 4 mm).

The saturated water absorption was determined according to GB/T 1034-2008. Injection-molded dumbbell specimens were dried at 120 °C for 4 h, then immersed in deionized water at room temperature for 30 days. Samples were periodically removed, surface-dried, and weighed.

## 3. Results and Discussion

### 3.1. Chemical Structure Analysis

Figure S1 shows the structural characterization spectrum of synthesized CzIPA. Figure S1a,b characterize the nuclear magnetic hydrogen and carbon spectra of the prepared CzIPA, while Figure S1d characterize the infrared and mass spectra of the prepared CzIPA. The results indicate that the chemical structure of the prepared dicarboxylic acid conforms to the expected structural characteristics.

**Figure 2:**
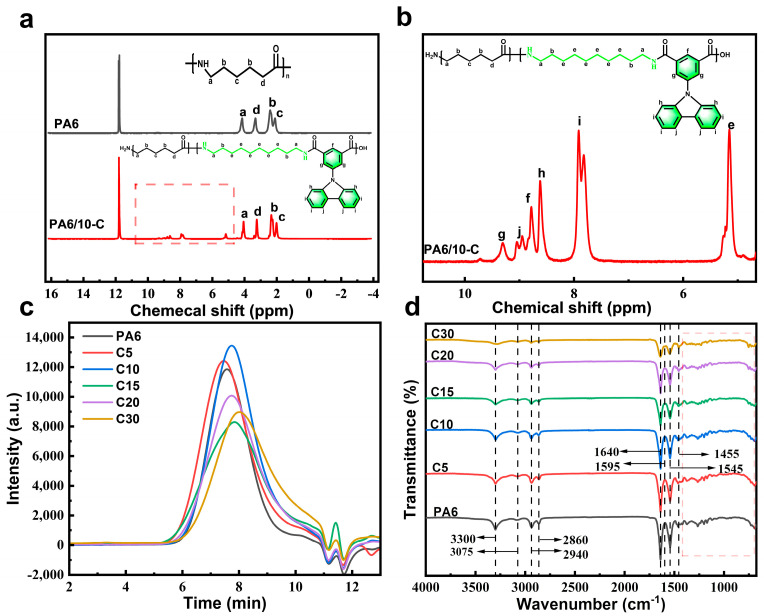
PA6 and copolymer (**a**) NMR hydrogen spectrum (**b**) Detailed diagram (**c**) GPC curves (**d**) FTIR spectra.

The chemical structures of the copolymers were further confirmed by ^1^H NMR spectroscopy, as shown in Figure 2a,b. Compared with neat PA6, the spectra of the copolymers exhibit two sets of weak signals at approximately 5.0 ppm and 7.8–8.0 ppm, corresponding to the aromatic protons on the phenyl rings of the CzIPA units. In addition, a series of small peaks observed in the range of 8.6–9.3 ppm are assigned to the hydrogen atoms on the carbazole rings. Apart from the characteristic peaks of amide protons, these newly appearing aromatic signals provide direct evidence for the successful incorporation of carbazole-containing CzIPA segments into the polyamide backbone.

The molecular weights of the polymers determined by GPC are shown in Figure 2c and Figure S2. The synthesized polymers exhibit number-average molecular weights ($\bar{M_{n}}$) of approximately 10,000 and weight-average molecular weights ($\bar{M_{w}}$) of around 20,000, with molecular weight distribution ($\bar{M_{w}}/\bar{M_{n}}$) values close to 2.0. These results confirm the successful synthesis of high-molecular-weight polyamide copolymers with relatively narrow molecular weight distributions. Furthermore, the comparable molecular chain lengths across the series suggest good uniformity, which helps to minimize potential experimental deviations in subsequent analyses arising from molecular weight differences.

The FTIR spectra of the copolymers are shown in Figure 2d. A broad absorption band at 3300 cm^−1^ corresponds to the N–H stretching vibration of the amide groups. The peaks at 2850–2950 cm^−1^ are assigned to the C–H stretching vibrations of the methylene groups, while the peak at 3050 cm^−1^ arises from the aromatic C–H stretching of the carbazole moiety. The strong absorption at 1640 cm^−1^ is attributed to the C=O stretching vibration of the amide groups, and the band around 1540 cm^−1^ originates from the coupling of C–N stretching and N–H bending vibrations. In addition, a weak band at 1465 cm^−1^ corresponds to the C–H bending vibration of methylene groups, while several small peaks observed in the range of 1400–1500 cm^−1^ are associated with the C=C stretching vibrations of the carbazole aromatic ring. In the fingerprint region, the peaks at 750–720 cm^−1^ and 830–810 cm^−1^ are attributed to the characteristic peaks of the carbazole fused ring structure in Figure S4. The appearance of these characteristic carbazole peaks, confirms the successful synthesis of the copolymers containing carbazole units [37,38].

### 3.2. Thermal Properties Analysis

**Figure 3:**
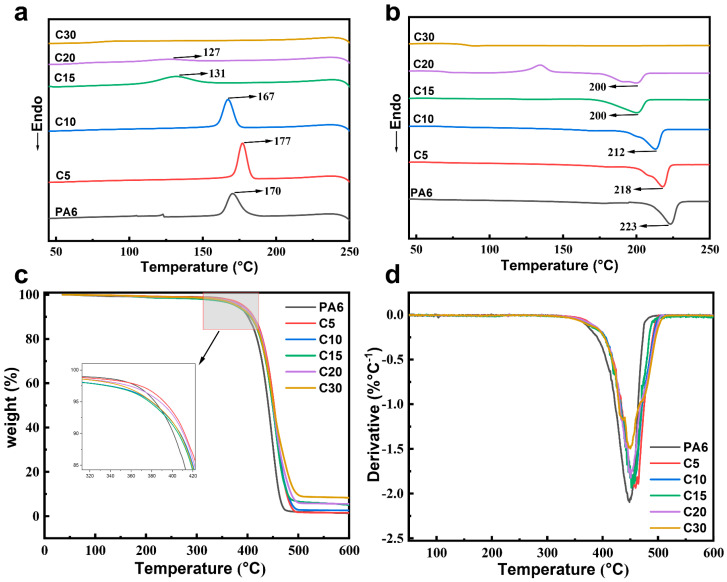
DSC and TG curves of PA6 and its copolymers (**a**) Cooling curves (**b**) Second heating curves (**c**) TG curves (**d**) DTG curves.

Figure 3a,b presents the DSC curves of PA6 and its copolymers. With the increasing incorporation of carbazole structural units, both the crystallization temperature (*T_c_*) and melting temperature (*T_m_*) of the copolymers gradually decrease, and the crystallization peaks become broader and less intense. This behavior indicates that the introduction of carbazole moieties disrupts the regularity and symmetry of the PA6 molecular chains. As the content of the imide-containing carbazole segments increases, the decline in *T_m_*, *T_c_*, and crystallization enthalpy becomes more pronounced. The bulky and rigid carbazole groups hinder chain mobility during cooling, impeding the orderly packing of macromolecular chains and resulting in reduced crystallinity [26]. Consequently, the incorporation of carbazole-based side-chain structures effectively suppresses crystal perfection and enhances the amorphous character of the copolymers.

Figure 3c,d displays the thermogravimetric (TG) and differential thermogravimetric (DTG) curves of PA6 and the PA6/10-Ccopolymers. As shown in the TG curves, all copolymers exhibit initial decomposition temperatures above 350 °C, indicating excellent thermal stability. The incorporation of carbazole-based aromatic structures significantly enhances the thermal resistance of the copolymers. With increasing content of the carbazole-containing monomer, the onset decomposition temperature (T_5%_) gradually rises, which can be attributed to the high thermal stability and conjugated rigidity of the carbazole and benzene ring structures. The DTG curves reveal that all samples display a single major degradation peak centered around 450 °C, confirming that the thermal degradation of these copolymers follows a one-step decomposition process. Overall, the introduction of the carbazole unit effectively improves the thermal stability of PA6, owing to its rigid aromatic backbone and enhanced char-forming capability at elevated temperatures [26].

**Figure 4:**
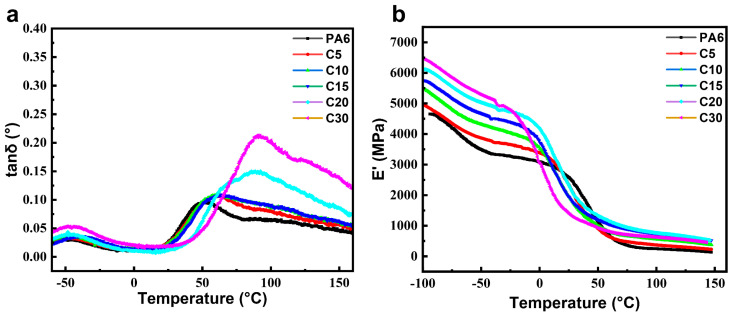
PA6 and copolymer (**a**) Energy storage modulus curve (**b**) Loss factor curve.

The dynamic mechanical analysis (DMA) was performed to investigate the variations of storage modulus (E′) and loss tangent (tan δ) of PA6 and the C-series copolymers with temperature. As shown in Figure 4, both the E′ and *T_g_* increased with the increasing content of carbazole-containing comonomer units. When the mass fraction of comonomer is 30%, *T_g_* is increased by about 35.5 °C compared to PA6; The tan δ curves exhibited two distinct relaxation peaks around −50 °C and 80 °C, corresponding to the β- and α-relaxations of the polyamide chains, respectively. The β-relaxation is typically associated with the local motion of non-hydrogen-bonded amide groups or side groups in the glassy state, resulting from internal rotation within the molecular chains. The α-relaxation, on the other hand, arises from the cooperative segmental motion in the amorphous regions and represents the glass transition process. The gradual increase in *T_g_* with higher carbazole unit content is attributed to the rigid aromatic structure of the carbazole moiety, which restricts the mobility of polymer chains and increases the activation energy required for segmental motion. Consequently, the incorporation of carbazole-containing comonomers enhances chain stiffness and thermal resistance in the copolymer system.

### 3.3. Crystallization Behavior Analysis

**Figure 5:**
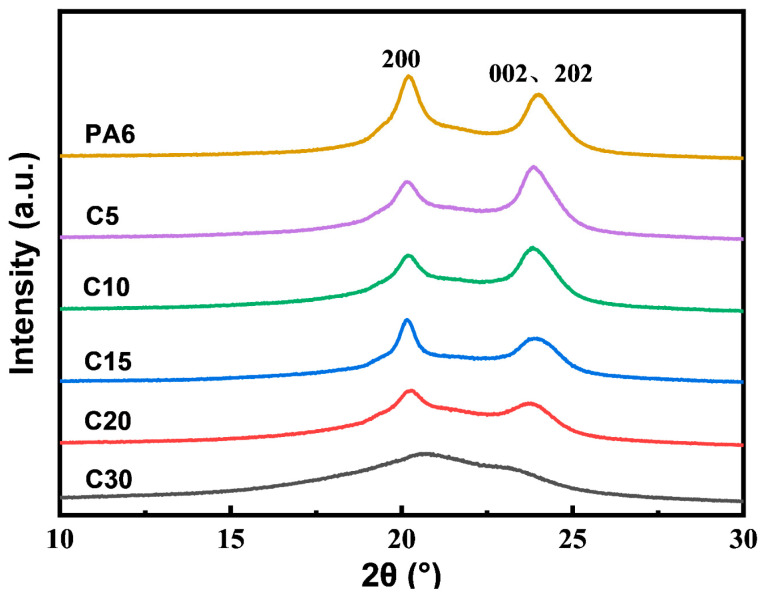
PA6 and copolymer XRD curve.

To further investigate the crystalline structures of neat PA6 and the copolymers, WAXD was used to analyze the crystalline structures of PA6 and the copolymers. As shown in Figure 5, both samples exhibit diffraction peaks at 2θ = 20° and 23.7°, corresponding to the (200) and (002)/(202) planes, indicating the presence of the *α*-crystalline phase. With increasing carbazole content, the diffraction peaks gradually broaden and weaken, while the amorphous halo becomes more pronounced, suggesting reduced crystallinity and increased disorder. In addition, the N–H stretching vibration bands in FTIR spectra shift to lower wavenumbers with higher carbazole incorporation, indicating weakened hydrogen bonding. These results confirm that the introduction of rigid carbazole units disrupts the regular chain packing of PA6, leading to a decrease in crystallinity and a more amorphous copolymer structure.

**Figure 6:**
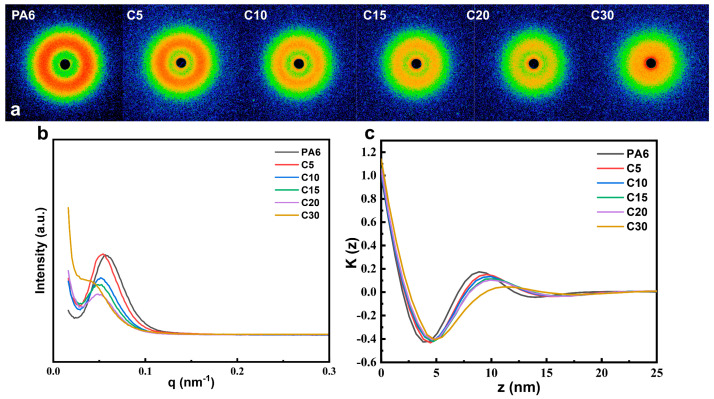
SAXS spectra of PA6 and its copolymer acyl groups. (**a**) Two-dimensional small-angle scattering patterns (**b**) SAXS one-dimensional integral curves (**c**) Lorenz calibration curve of SAXS one-dimensional integral curves.

Figure 6 presents the representative two-dimensional SAXS patterns of PA6 and a series of copolymer films containing various amounts of carbazole units. As the carbazole content increases, the overall scattering intensity gradually weakens, indicating a reduction in long-range lamellar order. Distinct scattering peaks appear around q = 0.05 nm^−1^ for all samples, suggesting the presence of periodic lamellar arrangements. Based on the electron density correlation function, the long period (*Lp*), amorphous layer thickness (*La*), and crystalline lamella thickness (*Lc*) of PA6 and its copolymers were calculated, as summarized in Figure S2. The copolymers exhibit smaller *Lc* values compared to neat PA6, primarily due to an increase in *La*, indicating that the introduction of bulky carbazole units disrupts the regular lamellar stacking and reduces the overall degree of crystallinity. These findings are consistent with WAXD results, further confirming that carbazole incorporation hinders crystal growth and promotes a more amorphous morphology.

### 3.4. Rheological Property Analysis

**Figure 7:**
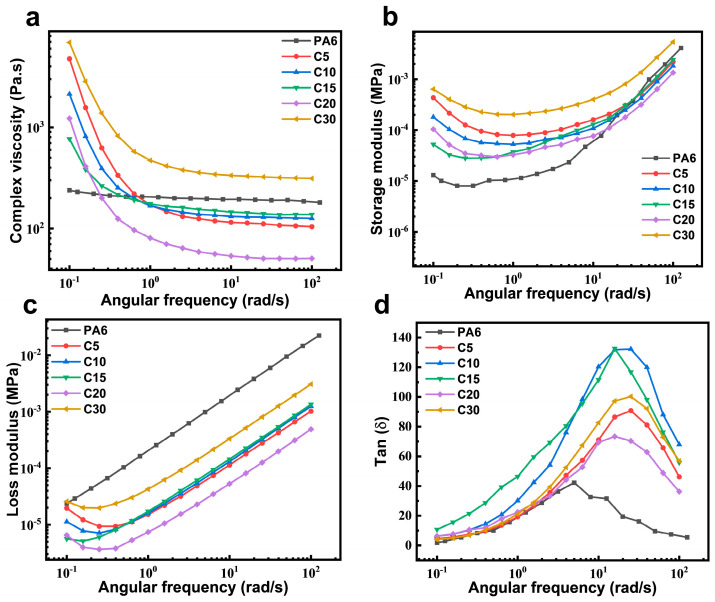
Rheological parameters of PA6 and its copolymer at 250 °C: (**a**) loss modulus, (**b**) storage modulus, (**c**) loss factor, (**d**) tan(δ).

Figure 7 shows the frequency sweep curves of the copolymers with different compositions. All samples exhibit typical linear viscoelastic behavior, with both the storage modulus (G′) and loss modulus (G″) increasing as the angular frequency rises. The G″ values remain higher than G′ across the entire frequency range, indicating that the copolymers are more viscous than elastic. At low frequencies, the copolymers display higher G′ values than neat PA6, suggesting enhanced elasticity due to restricted chain mobility. However, as the frequency increases, the growth rate of G′ for the copolymers becomes slower, and eventually G′ falls below that of PA6, reflecting reduced elasticity at high frequencies. The incorporation of rigid carbazole-containing CzIPA units limits chain segment motion, leading to constrained viscous flow.

Consequently, the copolymers show lower loss moduli at low frequencies, but both moduli increase with frequency. The larger rise in G″ compared to G′ indicates that molecular relaxation lags behind the applied deformation, resulting in an increased loss factor. At higher frequencies, the polymer chains are unable to respond promptly to the oscillation, leading to a “frozen” segmental state and a subsequent decline in the loss factor [39]. Furthermore, the complex viscosity (η*) decreases with increasing frequency, showing a pronounced shear-thinning behavior. This is attributed to molecular chain orientation along the shear direction under external forces. With higher CzIPA content, the shear-thinning effect becomes more prominent, as the disrupted crystallinity facilitates easier chain orientation during deformation.

### 3.5. Mechanical Property Analysis

**Figure 8:**
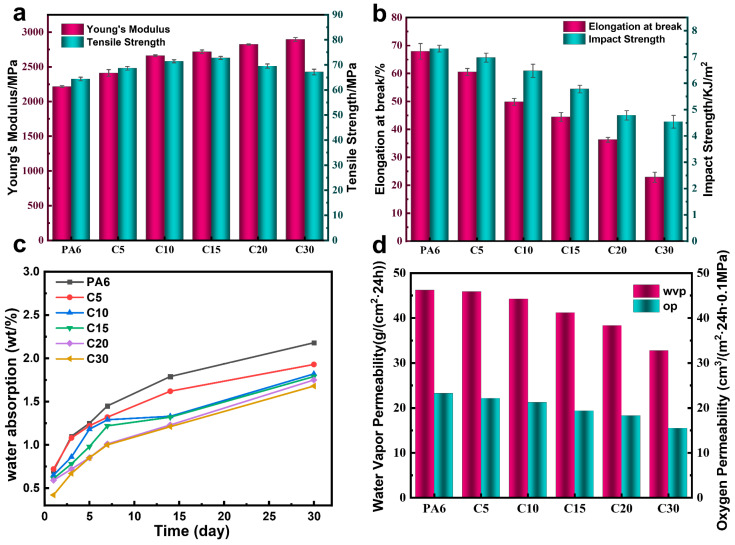
PA6 and Copolymers (**a**) Young’s modulus and Tensile strength. (**b**) Elongation at break and Impact strength (**c**) Water absorption rate (**d**) barrier performance.

Figure 8a,b and Figure S3 present the mechanical properties of neat PA6 and its CzIPA-based copolymers. The Young’s modulus increases progressively with the CzIPA content, while the elongation at break decreases, the tensile strength initially rises and then slightly declines at higher CzIPA loadings. However, at 30 wt% CzIPA, it remains higher than that of neat PA6. This behavior is attributed to the incorporation of rigid carbazole-containing aromatic rings, which enhance chain stiffness and restrict molecular motion, thereby increasing rigidity but reducing chain flexibility. The impact strength of the copolymers decreases with higher CzIPA incorporation, indicating reduced toughness and increased brittleness. Meanwhile, both the flexural strength and flexural modulus increase with the CzIPA content, consistent with the transition of the material from a ductile to a more brittle character. Overall, the introduction of rigid CzIPA units improves the stiffness and strength of PA6 but compromises its ductility, reflecting the trade-off between rigidity and toughness inherent to carbazole-based copolymer systems.

### 3.6. Dimensional Stability and Barrier Performance Analysis

Figure 8c illustrates the water absorption of PA6 and the copolymer. All samples display a rapid uptake at the early stage, followed by a gradual approach to equilibrium with increasing immersion time. This behavior is characteristic of polyamides and is governed by the strong polarity of the amide groups, which readily form hydrogen bonds with water molecules. Consequently, the initial diffusion is fast, while the subsequent increase becomes progressively slower as the available hydrogen-bonding sites become saturated. A reduction in equilibrium water absorption is observed with increasing incorporation of the comonomer. This trend can be attributed to the larger molecular size and bulkier structure of the introduced monomer, which effectively decreases the density of amide groups along the polymer backbone. The reduced concentration of hydrogen-bonding sites limits water—polymer interactions and thereby suppresses moisture uptake in the copolymer.

Figure 8d compares the oxygen transmission rate and water vapor transmission rate of neat PA6 and its CzIPA-based copolymers. Both oxygen transmission rate and water vapor transmission rate of decrease progressively with increasing copolymer content, indicating that incorporation of CzIPA units significantly enhances the barrier performance of PA6. Specifically, neat PA6 exhibits the highest WVTR of approximately 45 g/(m^2^·24 h), which decreases to about 33 g/(m^2^·24 h) at 30 wt% CzIPA content—representing a reduction of nearly 25%. Similarly, the OTR decreases from 23 cm^3^/(m^2^·24 h·0.1 MPa) for neat PA6 to approximately 15 cm^3^/(m^2^·24 h·0.1 MPa) for the C30 copolymer, corresponding to a 35% reduction. These results demonstrate that the introduction of rigid carbazole-containing CzIPA units effectively restricts chain mobility and optimizes the crystalline-amorphous morphology, thereby strengthening intermolecular interactions and extending the diffusion pathways for gas molecules.

## 4. Conclusions

A series of Carbazolyl copolymers were successfully synthesized by introducing CzIPA units into the PA6 backbone. The incorporation of rigid carbazole structures slightly reduced crystallinity but significantly enhanced the glass transition temperature, thermal stability, and dimensional stability of PA6. When the carbazole groups’ content reached 30 wt%, the *T_g_* increased by 35.5 °C and the maximum decomposition temperature by 23.8 °C compared with neat PA6. The Young’s and flexural moduli improved by about 13.1%, while water absorption decreased by 15.2%. Moreover, the copolymer exhibited a marked enhancement in barrier performance, with water vapor and oxygen transmission rates reduced by approximately 27% and 35%, respectively. Overall, this work demonstrates that copolymerization with carbazole monomers is an effective strategy to develop PA6 materials with superior rigidity, thermal resistance, and gas barrier properties for high-performance engineering and packaging applications.

## Data Availability

The original contributions presented in this study are included in the article/Supplementary Material. Further inquiries can be directed to the corresponding authors.

## Supplementary Materials

The following supporting information can be downloaded at: https://www.mdpi.com/article/10.3390/polym18050559/s1, Figure S1: Characterization of the chemical structure of synthesized CzIPA. (a) 1H-NMR, (b) 13C-NMR, (c) FTIR, (d) MALDI-TOF MS; Figure S2: PA6 and copolymer of Mn and PDI curve; Figure S3: Bending strength and Bending modulus of PA6 and the copolymers; Figure S4: Infrared spectra of PA6 and its copolyamides in the fingerprint region; Table S1: Raw material ratio of PA6/10-C resin.
