# Supplementary material for: Thermal Stability and Barrier Properties of Polyamide 6 Reinforced by Carbazole Based Copolymerization
Source: Polymers (Basel). 2026 Feb 25;18(5):559. doi: 10.3390/polym18050559 (PMC12986835; doi:10.3390/polym18050559)
Supplement: Supplementary file 1 [file polymers-18-00559-s001.zip › polymers-4145780-supplementary.pdf]

Supplementary Materials

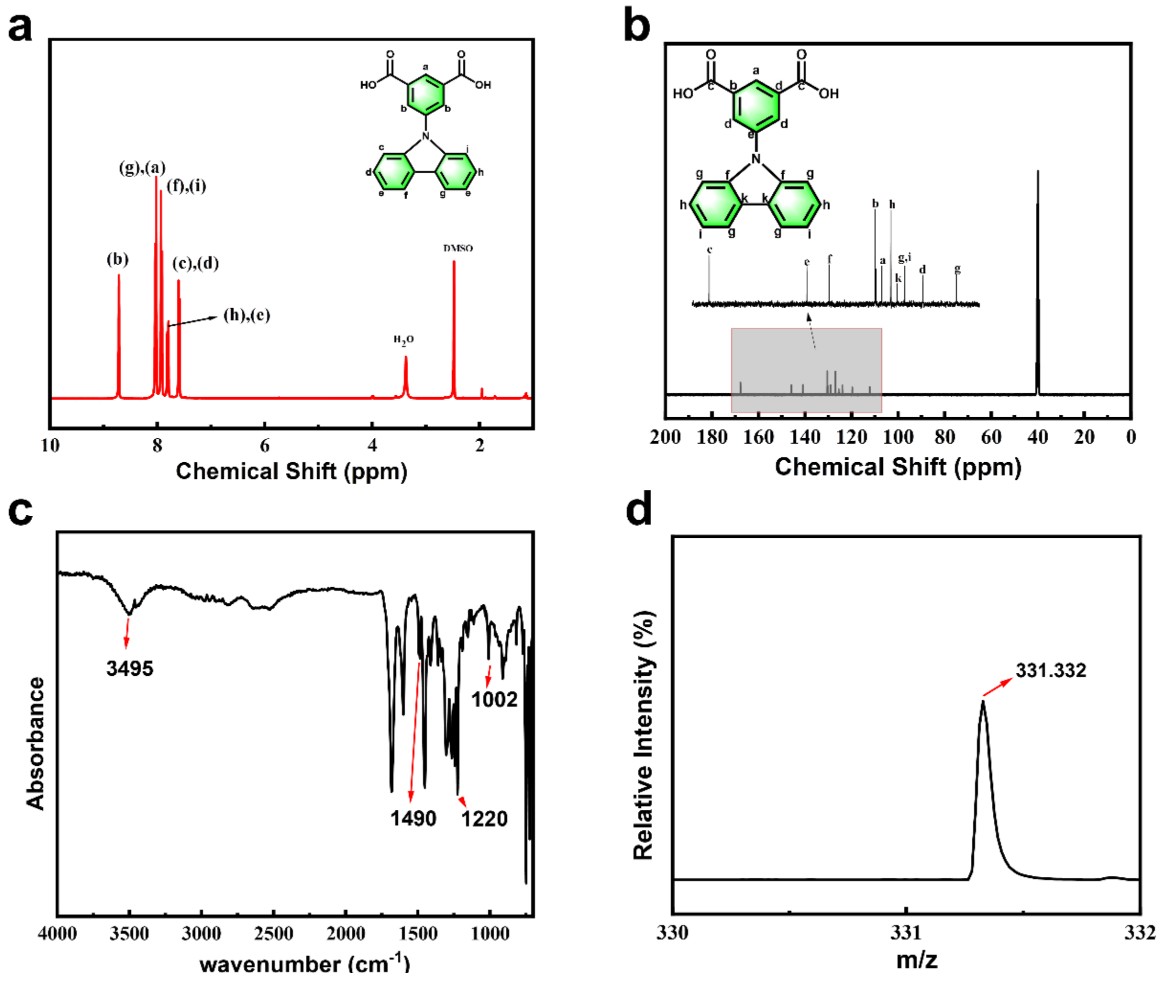

Figure S1. Characterization of the chemical structure of synthesized CzIPA. (**a**) 1H-NMR, (**b**) 13C-NMR, (**c**) FTIR, (**d**) MALDI-TOF MS.

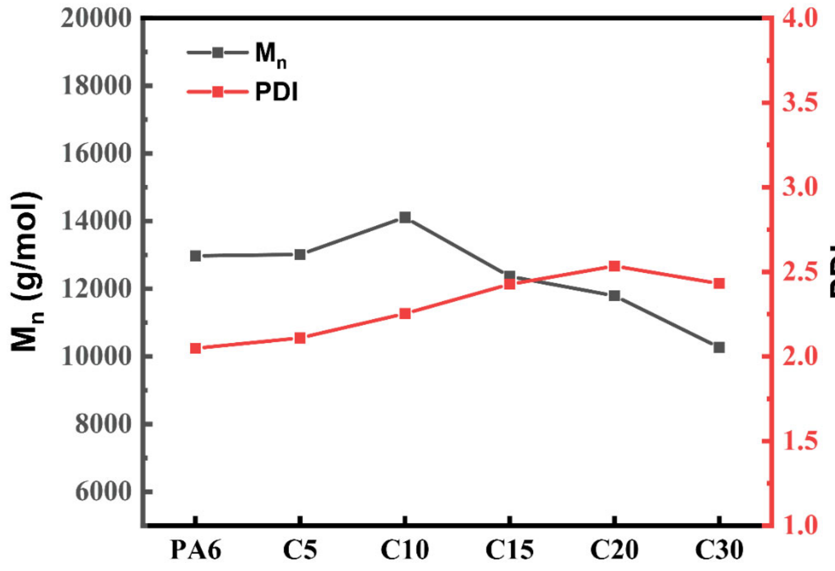

Figure S2. PA6 and copolymer of M_n and PDI curve.

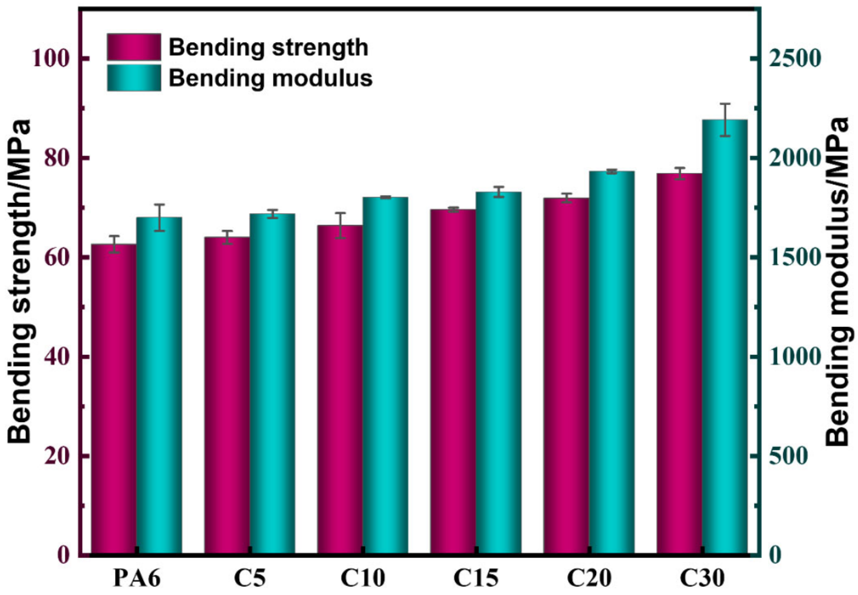

Figure S3. Bending strength and Bending modulus of PA6 and the copolymers.

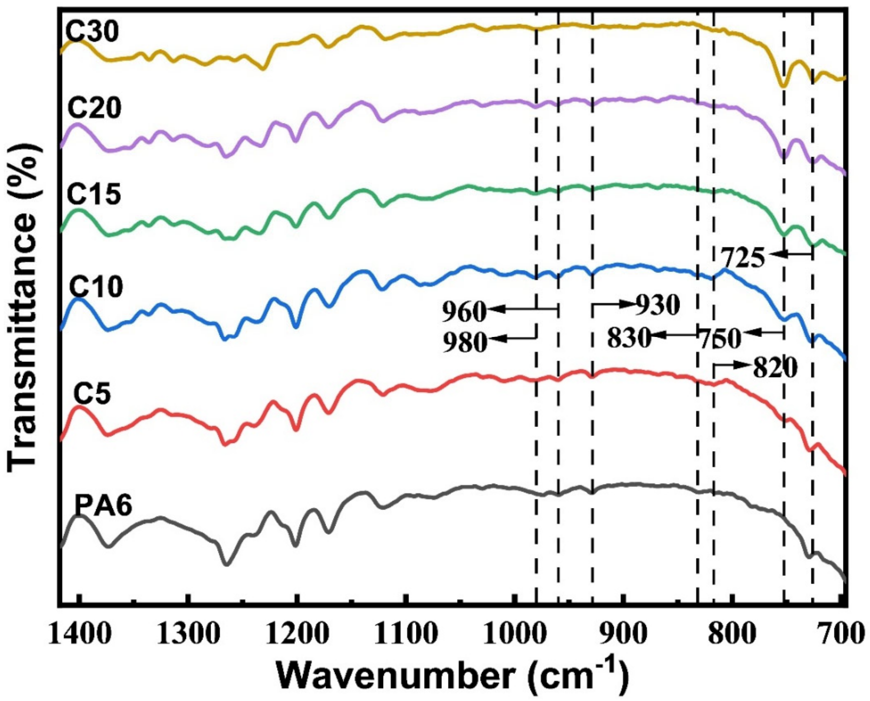

Figure S4. Infrared spectra of PA6 and its copolyamides in the fingerprint region.

Table S1. Raw material ratio of PA6/10-C resin.

Samples	CzIPA/g	Diamine /g	CPL/g	Mass Fraction of Nylon Salt /%
PA6	0	0	4000	0
C5	140.56	59.44	3800	5
C10	281.12	118.88	3600	10
C15	421.68	178.32	3400	15
C20	562.23	237.77	3200	20
C30	843.35	356.65	2800	30
